# Genomic characterization of aggressiveness in pituitary neuroendocrine tumors

**DOI:** 10.1093/neuonc/noag074

**Published:** 2026-08-06

**Authors:** Nesrine Benanteur, Chiara Villa, Fabio Bioletto, Maria Francesca Birtolo, Daniel De Murat, Julien Masliah-Planchon, Victor Gravrand, Inès Lebib, Mounia Benkhellat, Robin Boezennec, Stéphanie Allassonnière, Franck Letourneur, Yoann Martin, Fidéline Bonnet Serrano, Julian Jacob, Valentin Calugaru, Maxime Barat, Anthony Dohan, Jennifer Arrondeau, Olivier Huillard, Marie-Laure Raffin-Sanson, Jean François Emile, Gérald Raverot, Rossella Libé, Laurence Guignat, Cyril Garcia, Lionel Groussin, Xavier Bertagna, Stephan Gaillard, Jérôme Bertherat, Anne Jouinot, Bertrand Baussart, Guillaume Assié

**Affiliations:** CNRS UMR8104, INSERM U1016, Institut Cochin, Université Paris Cité, Paris, France; CNRS UMR8104, INSERM U1016, Institut Cochin, Université Paris Cité, Paris, France; Department of Pathology, Hôpital Lariboisière, Assistance Publique-Hôpitaux de Paris, Paris, France; CNRS UMR8104, INSERM U1016, Institut Cochin, Université Paris Cité, Paris, France; Division of Endocrinology, Diabetes and Metabolism, Department of Medical Sciences, University of Turin, Turin, Italy; CNRS UMR8104, INSERM U1016, Institut Cochin, Université Paris Cité, Paris, France; Department of Biomedical Sciences, Humanitas University, Milan, Italy; CNRS UMR8104, INSERM U1016, Institut Cochin, Université Paris Cité, Paris, France; Department of Diagnostic and Theranostic Medicine, Somatic Genetics Unit, Institut Curie, Paris-Science Lettres University, Paris, France; CNRS UMR8104, INSERM U1016, Institut Cochin, Université Paris Cité, Paris, France; CNRS UMR8104, INSERM U1016, Institut Cochin, Université Paris Cité, Paris, France; CNRS UMR8104, INSERM U1016, Institut Cochin, Université Paris Cité, Paris, France; CNRS UMR8104, INSERM U1016, Institut Cochin, Université Paris Cité, Paris, France; Université Paris Cité, Unité Mixte de Recherche S1138, Institut National de Recherche en Sciences et Technologies du Numérique, Sorbonne University, Paris, France; INSERM U1016, Institut Cochin, Plate-Forme Séquençage et Génomique (Genom’IC), Paris, France; CNRS UMR8104, INSERM U1016, Institut Cochin, Université Paris Cité, Paris, France; INSERM U1016, Institut Cochin, Plate-Forme Séquençage et Génomique (Genom’IC), Paris, France; CNRS UMR8104, INSERM U1016, Institut Cochin, Université Paris Cité, Paris, France; Hormonology Department, Cochin Hospital, Paris, France; Department of Radiation Oncology, Hôpital La Pitié-Salpêtrière, Assistance Publique-Hôpitaux de Paris, Paris, France; Department of Radiation Oncology, Paris/Saint-Cloud/Orsay, Institut Curie, PSL Research University, Paris, France; CNRS UMR8104, INSERM U1016, Institut Cochin, Université Paris Cité, Paris, France; Department of Radiology, Hôpital Cochin, Assistance Publique-Hôpitaux de Paris, Paris, France; CNRS UMR8104, INSERM U1016, Institut Cochin, Université Paris Cité, Paris, France; Department of Radiology, Hôpital Cochin, Assistance Publique-Hôpitaux de Paris, Paris, France; Department of Clinical Oncology, Hôpital Cochin, Assistance Publique-Hôpitaux de Paris, Paris, France; Medical Oncology Department, Université Paris Cité, Institut du Cancer Paris CARPEM, AP-HP, Hôpital Cochin-Port Royal, Paris, France; Department of Endocrinology, Diabetology, and Nutrition, Ambroise Paré University Hospital, Assistance Publique-Hôpitaux de Paris, Boulogne Billancourt, France; EA4340, University of Versailles Saint-Quentin-en-Yvelines, UFR of Health Sciences Simone Veil, Montigny-le-Bretonneux, France; Department of Pathology, Hôpital Ambroise Paré, Assistance Publique-Hôpitaux de Paris, Boulogne-Billancourt, France; Cancer Research Center of Lyon, Inserm U1052, CNRS UMR5286, Lyon 1 University, Lyon, France; Endocrinology Department, Reference Center for Rare Pituitary Diseases HYPO, “Groupement Hospitalier Est” Hospices Civils de Lyon, Bron, France; CNRS UMR8104, INSERM U1016, Institut Cochin, Université Paris Cité, Paris, France; Department of Endocrinology, Hôpital Cochin, Reference and Competence Center for Rare Adrenal Diseases and Rare Pituitary Diseases, Assistance Publique-Hôpitaux de Paris, Paris, France; Department of Endocrinology, Hôpital Cochin, Reference and Competence Center for Rare Adrenal Diseases and Rare Pituitary Diseases, Assistance Publique-Hôpitaux de Paris, Paris, France; Service D’Endocrinologie, Hôpital National D’Instruction des Armées Bégin, Saint-Mandé, France; CNRS UMR8104, INSERM U1016, Institut Cochin, Université Paris Cité, Paris, France; Department of Endocrinology, Hôpital Cochin, Reference and Competence Center for Rare Adrenal Diseases and Rare Pituitary Diseases, Assistance Publique-Hôpitaux de Paris, Paris, France; CNRS UMR8104, INSERM U1016, Institut Cochin, Université Paris Cité, Paris, France; Department of Endocrinology, Hôpital Cochin, Reference and Competence Center for Rare Adrenal Diseases and Rare Pituitary Diseases, Assistance Publique-Hôpitaux de Paris, Paris, France; CNRS UMR8104, INSERM U1016, Institut Cochin, Université Paris Cité, Paris, France; CNRS UMR8104, INSERM U1016, Institut Cochin, Université Paris Cité, Paris, France; Department of Endocrinology, Hôpital Cochin, Reference and Competence Center for Rare Adrenal Diseases and Rare Pituitary Diseases, Assistance Publique-Hôpitaux de Paris, Paris, France; CNRS UMR8104, INSERM U1016, Institut Cochin, Université Paris Cité, Paris, France; CNRS UMR8104, INSERM U1016, Institut Cochin, Université Paris Cité, Paris, France; Department of Neurosurgery, Hôpital Lariboisière, Assistance Publique-Hôpitaux de Paris, Paris, France; CNRS UMR8104, INSERM U1016, Institut Cochin, Université Paris Cité, Paris, France; Department of Endocrinology, Hôpital Cochin, Reference and Competence Center for Rare Adrenal Diseases and Rare Pituitary Diseases, Assistance Publique-Hôpitaux de Paris, Paris, France

**Keywords:** carcinoma, genomics, *LRP1B*, PitNETs, prognosis

## Abstract

**Background:**

Aggressive evolution of PitNETs is rare; metastatic spread is even more. Defining aggressiveness and malignancy is challenging, subsequently hard to predict, and to understand. The aim was to provide a molecular definition of aggressiveness using genomic approaches.

**Methods:**

PitNETs from 206 patients were included. Associations between 9 clinicopathological features of aggressiveness and PitNETs’ omics were explored. Omics included transcriptome, DNA methylation, chromosomal alterations, and mutations. Clonal tumor evolution was monitored in 7 patients.

**Results:**

Among the 9 clinicopathological features of aggressiveness, only rapid progression, progression after radiotherapy, Ki67/MIB1 proliferation index ≥10%, temozolomide treatment, metastases, and specific death were associated with specific omics signatures, while tumour maximal diameter ≥40 mm, cavernous, and sphenoid invasion were not. The omic signatures associated with these features of aggressiveness overlapped but remained distinct between corticotroph and mammo-somato-thyrotroph lineages. For each lineage, a common signature of aggressiveness was identified, associating a proliferative transcriptome signature and DNA hypermethylation. Alterations in specific genes were associated with aggressive features, including a novel PitNET gene, *LRP1B*, and known cancer genes (*TP53*, *CDKN2A*), while *USP8* and *GNAS* alterations were not. Integration of gene alterations with methylome and transcriptome signatures isolated a subset of molecularly aggressive PitNETs. Molecular signatures were stable during the course of the disease, despite evolution toward aggressiveness and potential clonal divergence.

**Conclusion:**

This systematic analysis of clinicopathological features of aggressiveness using an integrated multiomic approach establishes a histomolecular definition of aggressiveness in PitNETs. Prospective cohort studies are needed to validate these molecular signatures and establish their prognostic value.

Key Points

- Definition of aggressiveness in PitNETs is debatable.
- Multiomic integration identifies distinct clusters of PitNETs with different outcomes.
- Combining these clusters with pathology, we propose a histomolecular risk stratification of aggressiveness/malignancy.

Importance of the Study

Clinical definitions of aggressiveness in PitNETs are hampered by the rarity of unfavorable evolutions, the heterogeneity of clinical managements, the limited standardization of classification and outcome criteria, and the confounding impact of therapeutics on outcome.

Here, we explored to which extent multimodal genomic classifications could improve the definition of aggressiveness. By testing common clinicopathological features of aggressiveness, we isolated those associated with a significant molecular signature. In parallel, unsupervised multimodal genomic classifications isolated molecular clusters of PitNETs with an unfavorable outcome. Notably, these clusters were associated with the significant clinicopathological features of aggressiveness. From these clusters, corticotroph and mammo-somato-thyrotroph signatures of aggressiveness were established. Longitudinal genomic screening identified common subclonal divergence in PitNETs with an unfavorable outcome. However, the associated signatures of aggressiveness remained stable.

Combining the molecular signatures with histopathological features, we proposed a pragmatic histomolecular risk assessment, which could be implemented in clinical routine to better predict unfavorable evolution.

Pituitary Neuroendocrine Tumors (PitNETs) are common.^1^ The clinical presentation of PitNETs ranges from indolent tumors to locally invasive, up to resistant, multi-recurrent, or even aggressive tumors, with or without metastatic spread. The clinical impact is determined by secretion type, tumor burden, and disease control.^2^ Metastatic PitNETs are very rare (∼0.2%).^3^

PitNETs “aggressiveness” is debatable.^4^ Clinically, it may include large size, invasion, rapid growth, therapy resistance, progression after radiotherapy, temozolomide, metastases, and death. Pathologically, it may be linked to high proliferation and lactotroph and corticotroph subtypes (TPIT and PIT1 lineages, respectively),^5–7^ though prognostic value is limited.^8^ Molecularly, aggressiveness has been associated with alterations in cell cycle (*TP53*, *CDKN2A*, *RB1)*, DNA repair (*MLH1*, *MSH6)*, chromatin structure (*ATRX*, *DAXX*, *MEN1*), and splicing genes (*SF3B1*).^9–12^  *USP8* and *GNAS* mutations are common but of debated prognostic value.^13^^,^^14^ Transcriptome and DNA methylation profiles have been reported in PitNETs.^15–17^ Signatures reflecting lineage were strong, more than the molecular variability associated with aggressiveness. This suggests that aggressiveness should be studied separately in each lineage.^15–17^ Recently, a DNA methylation signature of aggressiveness was proposed by comparing aggressive and metastatic PitNETs to non-aggressive tumors.^18^ An independent cohort would help to validate this signature and explore the interference with lineages. Two recent single-cell transcriptome studies reported a proliferative subpopulation of cells, associated with proliferation and aggressiveness.^19^^,^^20^

Here, transcriptome, methylome, and genomic alterations data were analyzed in 206 patients (134 from our previous study,^17^ and 72 with clinicopathological features of aggressiveness). Two approaches were combined. Starting from clinicopathological features of aggressiveness, we identified those associated with a genomic signature. Starting from the genomic profiles of tumors, we identified subgroups of PitNETs accumulating these clinicopathological features of aggressiveness. Integrating these, we identified molecularly defined aggressive PitNETs and explored signature dynamics over disease progression.

## Methods

### Patients Samples

Two-hundred and 6 patients with 228 PitNETs were included, representing all subtypes. Clinical, pathological, and hormonal exploration were performed following standard recommendations.^21^

A pituitary MRI was performed for all patients before surgery. Post-surgical MRI follow-up was repeated following clinical guidelines.^21^ MRIs were reviewed by an expert radiologist (MB).

All patients were operated on between 1997 and 2024. Patients underwent 1 to 5 PitNET surgeries. The latest tumor available for each patient was used for transcriptome, methylome, and gene alteration classifications. The latest available sample corresponded to a first, second, and third or later surgery for 183 (89%), 6 (3%), and 17(8%) patients, respectively. For 14 patients, multiple samples were available (2 to 5) for longitudinal analyses. All tumor samples were reviewed by an expert pathologist (CV). Histological subtype was defined according to the WHO 2025 classification of endocrine tumors.^6^ Anterior pituitary hormones expression (ACTH, PRL, GH, FSH, LH, TSH), transcription factors (TPIT, PIT1, SF1, GATA3), granulation, and Ki67 (MIB1) were assessed by immunochemistry using a fully automated IHC slide staining instrument, BenchMark XT (Roche—Ventana Medical System, Inc., Tucson, AZ, USA).

A total of 41 patients received radiotherapy. Post-radiotherapy pituitary MRI was performed over a 6-month follow-up period. In view of the possible latency of radiotherapy effects, cases showing initial tumor enlargement on the first post-radiotherapy MRI, followed by a sustained phase of tumor stability or regression without further tumor-directed intervention, were not classified as cases of tumor progression.

Twenty-one patients received temozolomide.

### Clincal Features of Aggressiveness

Nine clinicopathological features of aggressiveness were collected for all patients, including: (i) tumor maximal diameter ≥40 mm; (ii) cavernous invasion, and (iii) sphenoid invasion. Cavernous invasion was scored according to the Knosp grading system.^22^ Sphenoidal invasion was reported when visible on MRI; (iv) Ki67 ≥ 10%. The MIB1/Ki67 labeling index was measured in 2 hotspots (500 and 1000 tumour cells per hotspot), and all labeled tumour cells were counted regardless of intensity. The number of positive nuclei was expressed as the percentage (index) of the overall number of tumour cells.^23^^,^^24^ The antibody features and the experimental conditions are listed in Table S1; (v) progression after radiotherapy. To ascertain progression, a call was made when *a *≥ 2 mm increase of the 3 dimensions of the tumor was observed, in association with at least 1 of the 3 following features: (1) increase in pituitary stalk deviation; (2) increase in the upward convexity of the diaphragma sellae; or (3) increase in sellar floor asymmetry^25^^,^^26^; (vi) rapid progression. Tumor progression speed was defined as maximal diameter variation divided by the time between 2 consecutive MRIs. Rapid progression was called in 15 patients for diameter variations ranging from 10 to 48 (median 16) mm per year; (vii) temozolomide treatment, irrespective of the response to treatment; (viii) metastases; (ix) specific death.

### Transcriptome Analyses

Transcriptome data for 227 PitNETs were analyzed, including 134 from the previously published series by Neou et al,^17^ and 93 additional PitNETs enriched in features of aggressiveness. PitNETs transcriptome was generated from either frozen or Formalin-Fixed and paraffin-embedded (FFPE) samples (Figure S1A). RNA sequencing was generated using TruSeq Stranded mRNA protocol (Illumina) for frozen samples and QuantSeq 3′ mRNA-Seq protocol (Lexogen) for FFPE sample.s Detailed protocols and bioinformatic methods are provided as Supplementary Methods. Tumor cells content was assessed by deconvoluting the signatures of each lineage (Figure S1B).

### Methylome Analyses

Methylome data for 100 PitNETs were analyzed, including 86 from the previously published series by Neou et al,^17^ and 14 additional PitNETs exhibiting features of aggressiveness. Whole-genome DNA methylation was performed using the Infinium MethylationEPIC BeadChip (Illumina). Detailed protocols and bionformatic methods are provided as Supplementary Methods.

### Gene Alterations Landscape Analyses

Sequencing data for 156 PitNETs were analyzed, including 114 from the previously published series by Neou et al,^17^ and 42 additional PitNETs exhibiting features of aggressiveness. 144 of these tumors were sequenced using exome analysis and/or RNA-based inference, while 12 underwent targeted panel sequencing. Copy number variation was explored by SNP array using the Genome Alteration Print tool^27^ in 86 patients from the previously published series by Neou et al^17^ and 21 additional PitNETs exhibiting features of aggressiveness. Tumor cell content was assessed by the allelic ratios of chromosomal alterations (Figure S1C).

## Results

### Patients

A cohort of PitNETs from 206 patients enriched in all histotypes and in features of aggressiveness was characterized. Nine clinicopathological features of aggressiveness were explored, including tumor maximal diameter ≥40 mm (*N*  =  19/198, 9.6%), cavernous invasion (*N*  =  110/204, 54%), sphenoid invasion (*N*  =  96/197, 48.7%), Ki67 ≥ 10% (*N*  =  37/203, 18.2%), progression after radiotherapy (*N*  =  21/206, 10%), rapid progression (*N*  =  14/206, 6.8%), temozolomide treatment (*N*  =  21/206, 10.2%), metastases (*N*  =  5/206, 2.4%), and specific death (*N*  =  13/206, 6.3%) (Table S2). Median follow-up after surgery was 28 months (IQR: 4-60 months).

### Transcriptome Signatures of Aggressiveness

**Figure 1.**
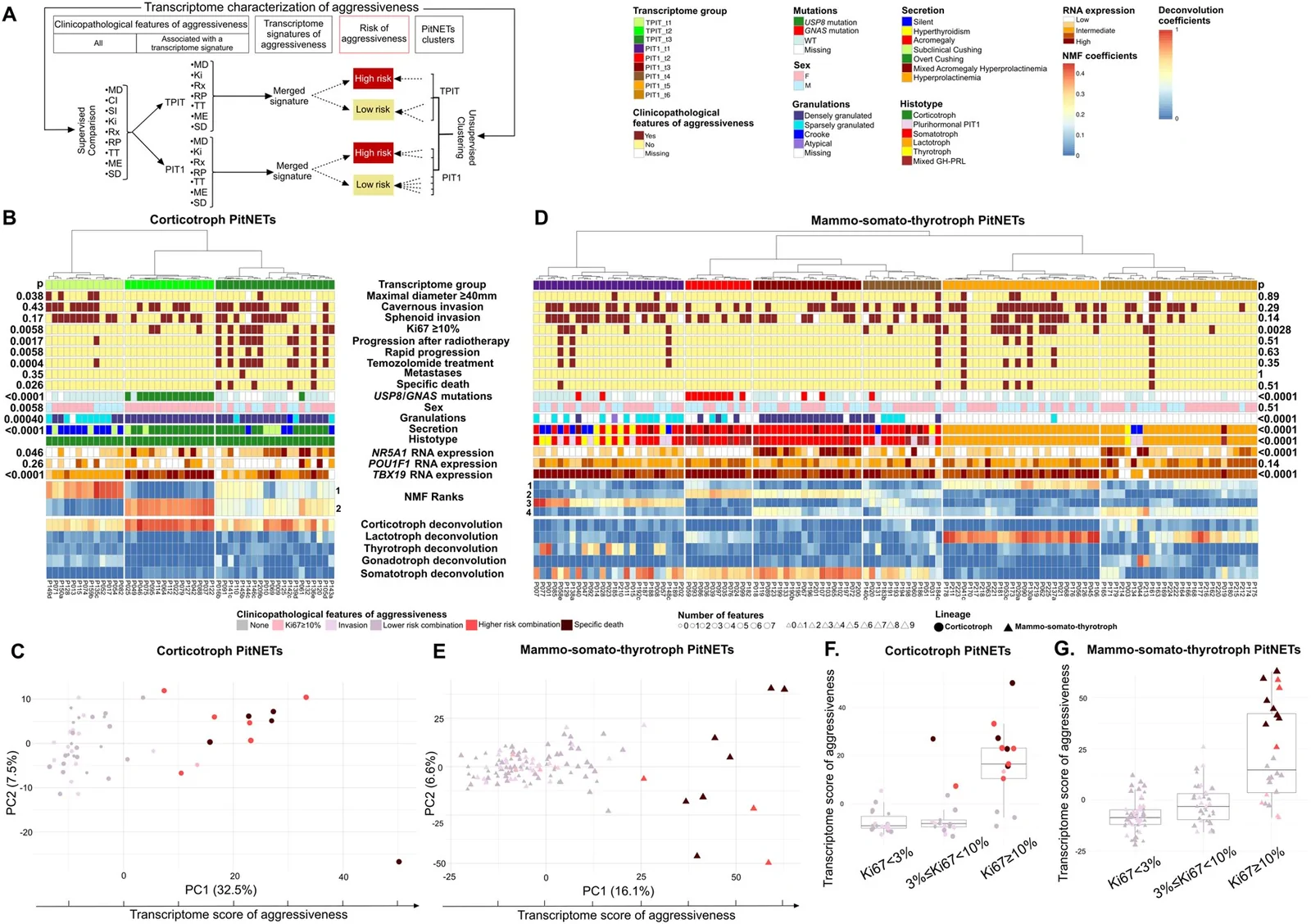
Transcriptome signatures of aggressiveness. (A) Flowchart of transcriptome analyses, combining supervised (left part) and unsupervised (right part) approaches. MD: maximal diameter ≥40 mm, CI: cavernous invasion, SI: sphenoid invasion, Ki: Ki67 ≥10%, Rx: progression after radiotherapy, RP: rapid progression, TT: temozolomide treatment, ME: metastases, SD: specific death. (B) Unsupervised classification of corticotroph PitNETs; the most robust partition was obtained with 2 NMF ranks and the 1500 most variable genes. (C) Transcriptome score of aggressiveness in corticotroph PitNETs, defined by the principal component 1 (PC1) coordinate of tumors on a dedicated PCA projection, based on the differentially expressed genes between PitNETs accumulating the significant features of aggressiveness and other PitNETs. (D) Unsupervised classification of mammo-somato-thyrotroph PitNETs; the most robust partition was obtained with 4 NMF ranks and the 1000 most variable genes. (E) Transcriptome score of aggressiveness in mammo-somato-thyrotroph PitNETs, defined by the principal component 1 (PC1) coordinate of tumors on a dedicated PCA projection, based on the differentially expressed genes between PitNETs accumulating the significant features of aggressiveness and other PitNETs. Boxplot representation in corticotroph (F) and in mammo-somato-thyrotroph (G) tumors of the transcriptome score of aggressiveness depending on Ki67/MIB1 proliferation index as defined by pathology. In 9 patients with PitNETs showing Ki67 ≥10%, transcriptome scores of aggressiveness were low (below the lower quartile of scores in the Ki67 ≥10% classes displayed on panels F and G for corticotroph and mammo-somato-thyrotroph respectively). Six of these patients were in remission (3 corticotroph tumors, 2 lactotroph tumors and 1 somatotroph tumor; median follow-up: 6 months, range: 1 to 30), and 3 presented a persistent disease controlled by standard therapy (2 lactotroph tumors under dopamine agonists, and 1 somatotroph tumor under somatostatin analogs; follow-up: 1, 1, and 74 months). Conversely, in 4 patients with PitNETs showing intermediate Ki67 (3 to <10%), transcriptome scores of aggressiveness were high (above the median of scores in the Ki67 ≥10% classes displayed on panels F and G for corticotroph and mammo-somato-thyrotroph, respectively). Three of these patients showed a persistent lactotroph tumor (all controlled by dopamine agonists but one requiring high doses; follow-up: 44, 50, and 108 months), and one cotricotroph tumor with an unfavorable outcome (specific death after 4 years). Accumulation of clinicopathological features of aggressiveness was coded in 6 categories: (i) none, for patients with no feature; (ii) Ki67 ≥10%, for patients with tumor Ki67 ≥10% and no additional feature of aggressiveness; (iii) invasion, for patients with tumor invasion (cavernous and/or sphenoid); (iv) lower risk combination, for patients with tumor Ki67 ≥10%, and invasion (cavernous and/or sphenoid), and/or tumor maximal diameter ≥40 mm; (v) higher risk combination, for patients with progression after radiotherapy and/or rapid progression, and/or temozolomide treatment, and/or metastases (in addition to tumor Ki67 ≥10%, and invasion, and/or tumor maximal diameter ≥40 mm); (vi) specific death.

Transcriptomes of 205/206 patients were analyzed using 2 approaches: unsupervised clustering to explore PitNETs classification, and supervised comparisons to identify signatures associated with each of the 9 clinicopathological features of aggressiveness (Figure 1A).

#### Unsupervised Transcriptome Classification (All Lineages)

Unsupervised clustering separated corticotroph, mammo-somato-thyrotroph, and gonadotroph PitNETs, reflecting lineage (Figure S2A, Table S3). Clinico­pathological features of aggressiveness (Ki67 ≥ 10%, progression after radiotherapy, rapid progression, temozolomide treatment, metastases, specific death) were enriched in subsets of corticotroph and mammo-somato-thyrotroph PitNETs, and absent in our cohort of gonadotroph and null-cell PitNETs (Figure S2A). Tumor maximal diameter ≥40 mm, sphenoid invasion, cavernous invasion, and metastases were not associated with any specific transcriptome cluster (Figure S2A). Of note, null-cell tumors clustered with gonadotroph PitNETs, as previously reported^17^ (Figure S2A).

#### Unsupervised Transcriptome Classification in the Corticotroph Lineage

Corticotroph PitNETs clustered in 3 groups as previously reported,^17^ referred to as TPIT_t1, TPIT_t2, and TPIT_t3 (Figure 1B, Table S4). Tumors with Ki67 ≥ 10%, progression after radiotherapy, rapid progression, temozolomide treatment, metastases, or specific death accumulated in TPIT_t3 (Figure 1B). TPIT_t3 was characterized by a high prevalence of males with overt Cushing syndrome, and a low prevalence of *USP8* mutation (Figure 1B). Of note, within TPIT_t3, 5/10 tumors with these clinicopathological features of aggressiveness presented apoplexy, and 2/10 a secretion shift from silent to functioning. Transcriptome signature of TPIT_t3 was enriched in genes related to translation (Tables S5 and S6).

TPIT_t1 and TPIT_t2 accumulated tumors with fewer clinicopathological features of aggressiveness (Figure 1B). TPIT_t1 was characterized by a high prevalence of non-functioning tumors (Figure 1B), of sparsely granulated tumors on pathology (Figure 1B), and by a corticotroph-gonadotroph signature, as previously reported^17^ (Figure 1B). Transcriptome signature of TPIT_t1 was enriched in genes related to cytoskeleton modification (Tables S5 and S6). TPIT_t2 was characterized by females with overt Cushing syndrome and *USP8* mutations (Figure 1B). Transcriptome signature of TPIT_t2 was enriched in genes related to synaptic transmission (Tables S5 and S6). Of note, cavernous invasion and sphenoid invasion were not associated with any specific corticotroph subgroup (Figure 1B).

#### Clinicopathological Features of Aggressiveness with Significant Transcriptome Signatures (Corticotroph Lineage)

We assessed the differential gene expression associated with each clinicopathological feature of aggressiveness by comparing PitNETs exhibiting the feature to those that did not. Significant transcriptome signatures were found for 7 clinicopathological features of aggressiveness, including tumor maximal diameter ≥40 mm, Ki67 ≥ 10%, progression after radiotherapy, rapid progression, temozolomide treatment, metastases, and specific death (Table S7). For cavernous invasion and sphenoid invasion, no specific signature was found.

A merged transcriptome signature of aggressiveness in corticotroph PitNETs was created by merging the transcriptome signatures associated with the 7 clinicopathological features of aggressiveness. This signature separated a subset of tumors accumulating the significant features of aggressiveness (Figure S2B, Table S7). In this subset of PitNETs, genes differentially expressed showed enrichment in proliferation (Figure S2C, Table S8). Using these differentially expressed genes, a transcriptome score of aggressiveness was built, corresponding to the principal component 1 (PC1) coordinate of each tumor on the PCA projection (Figure 1C).

#### Unsupervised Transcriptome Classification in the Mammo-Somato-Thyrotroph Lineage

Mammo-somato-thyrotroph PitNETs clustered in 6 groups, referred to as PIT1_t1 to PIT1_t6 (Figure 1D, Table S9). Specific signatures included translation in PIT1_t5 lactotroph group, and cilium and microtubule metabolism in both PIT1_t2 and PIT1_t3 somatotroph groups (Tables S10 and S11). Clinicopathological features of aggressiveness accumulated in PIT_t5 (5/21 tumors), but association with transcriptome groups was not significant (Fisher’s exact *P*  =  .22). Of note, 2 clusters accumulated less clinicopathological features of aggressiveness, including PIT1_t2—somatotroph with *GNAS* mutations and PIT1_t3—somatotroph co-expressing *NR5A1*/SF1 and *POU1F1*/PIT1 (Figure 1D).

#### Clinicopathological Features of Aggressiveness with Significant Transcriptome Signatures (Mammo-Somato-Thyrotroph Lineage)

We assessed the differential gene expression associated with each clinicopathological feature of aggressiveness by comparing PitNETs exhibiting the feature to those that did not. Significant transcriptome signatures were found for 7 clinicopathological features of aggressiveness, including tumor maximal diameter ≥40 mm, Ki67 ≥ 10%, progression after radiotherapy, rapid progression, temozolomide treatment, metastases, and specific death (Table S12). Conversely, for cavernous invasion and sphenoid invasion, the differential signature was limited to 3 and 2 genes, respectively (Table S12).

A merged transcriptome signature of aggressiveness in mammo-somato-thyrotroph PitNETs was created by merging the transcriptome signatures associated with the 7 clinicopathological features of aggressiveness. This signature separated a subset of tumors accumulating the significant features of aggressiveness (Figure S2D, Table S12). In this subset of PitNETs, genes differentially expressed showed enrichment in proliferation (Figure S2E, Table S13). Using these differentially expressed genes, a transcriptome score of aggressiveness was built, corresponding to the PC1 coordinate of each tumor on the PCA projection (Figure 1E).

#### Transcriptome Signatures of Proliferation in PitNETs

The differentially expressed genes in PitNETs accumulating clinicopathological features of aggressiveness reflected proliferation in both corticotroph and mammo-somato-thyrotroph PitNETs (Tables S8 and S13). However, these 2 signatures differed (Figure S2F and G), reflecting G1, S, and M cell cycle phases in corticotroph PitNETs, while restricted to M phase in mammo-somato-thyrotroph PitNETs (Tables S8 and S13 and Figure S2D and E).

We next explored the relation between transcriptome and pathology. In both corticotroph and mammo-somato-thyrotroph PitNETs, the transcriptome score of aggressiveness and Ki67/MIB1 proliferation index were associated (Kruskal *P* < .0001, Figure 1F and G). Discrepancies occurred in 9 patients with PitNETs showing Ki67 ≥ 10% and low transcriptome scores of aggressiveness (Figure 1F and G). Finally, focusing on PitNETs with Ki67 ≥ 10% and sphenoid or cavernous invasion without any of the other clinicopathological features of aggressiveness, 3/47 corticotroph and 10/119 mammo-somato-thyrotroph tumors showed a low transcriptome score of aggressiveness (Figure 1F and G).

We next inferred cell populations in each tumor from the bulk transcriptome, using PitNETs single-cell signatures recently reported.^19^^,^^20^^,^^28^ The contribution of each of these populations to the signatures of aggressiveness was assessed. Three of 38 published single cell signatures (T00_PTTG1,^19^ ACTH_Agg,^20^ TPIT_pro^20^) showed significant positive correlations with transcriptome scores in both corticotroph and mammo-somato-thyrotroph PitNETs (Tables S14 and S15 and Figure S2H and I).

### Methylome Signatures of Aggressiveness

**Figure 2.**
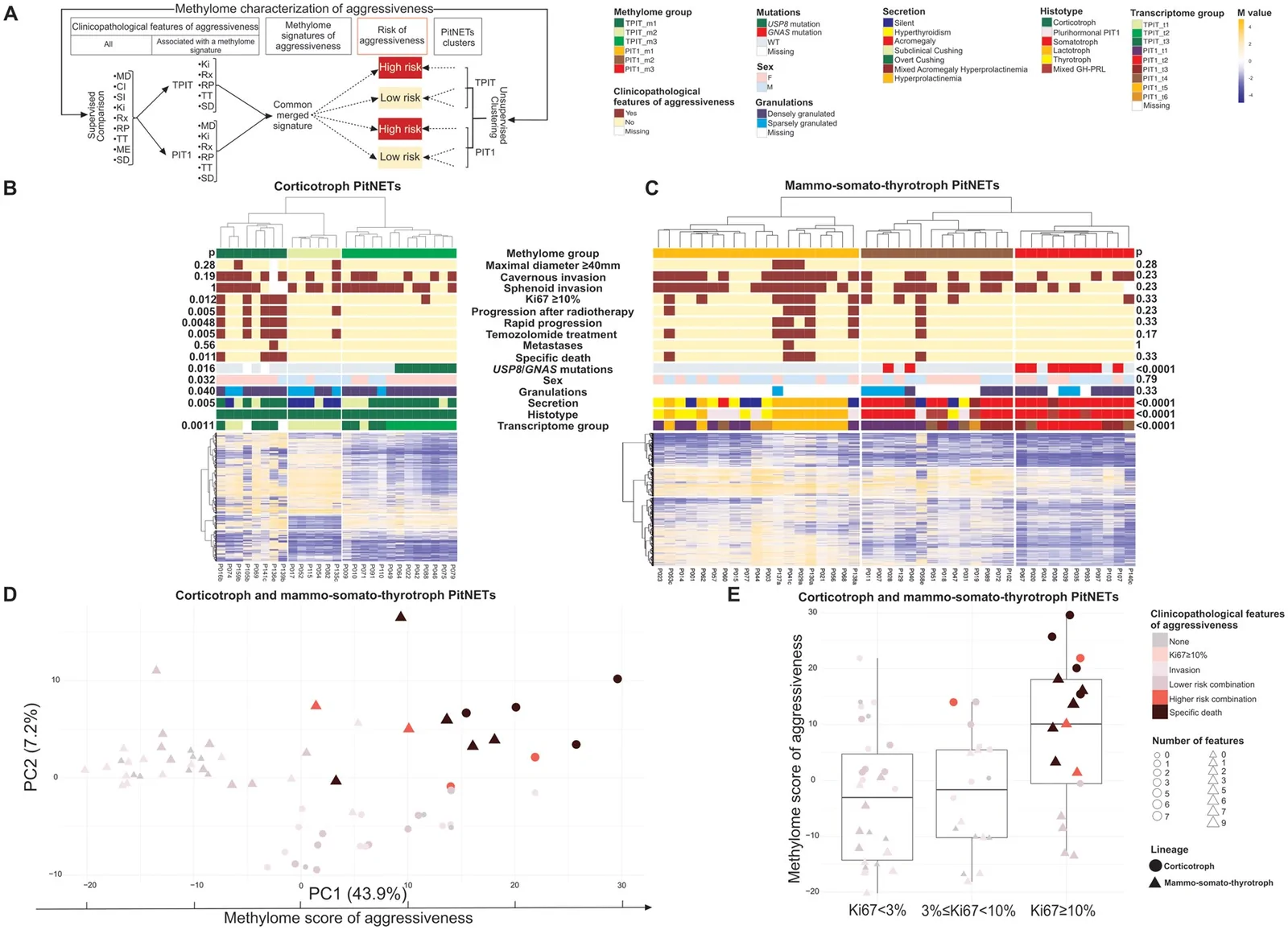
Methylome signatures of aggressiveness. (A) Flowchart of methylome analyses, combining supervised (left part) and unsupervised (right part) approaches. MD: maximal diameter ≥40 mm, CI: cavernous invasion, SI: sphenoid invasion, Ki: Ki67 ≥10%, Rx: progression after radiotherapy, RP: rapid progression, TT: temozolomide treatment, ME: metastases, SD: specific death. (B) Unsupervised classification of corticotroph PitNETs; the most robust partition obtained with 6000 most variable CpGs. (C) Unsupervised classification of mammo-somato-thyrotroph PitNETs; the most robust partition with 6000 most variable CpGs. (D) Methylome score of aggressiveness, defined by the principal component 1 (PC1) coordinate of corticotroph and mammo-somato-thyrotroph tumors on a dedicated PCA projection, based on the differentially expressed genes between PitNETs accumulating the significant features of aggressiveness and other PitNETs. (E) Boxplot representation in corticotroph and mammo-somato-thyrotroph tumors of the methylome score of aggressiveness depending on Ki67/MIB1 proliferation index as defined by pathology. In 4 patients with PitNETs showing Ki67 ≥10%, methylome scores of aggressiveness were low (below the lower quartile of scores in the Ki67 ≥10% class). One of these patients presenting a somatotroph tumor was in remission (follow-up: 6 months), and 3 showed a persistent disease (2 somatotroph tumors controlled by radiotherapy, somatostatin analogs, and dopamine agonists, and 1 thyrotroph tumor controlled by somatostatin analogs; follow-up: 5, 36, and 186 months). Conversely, in 3 patients with PitNETs showing intermediate Ki67 (3 to <10%), methylome scores of aggressiveness were high (above the median of scores in the Ki67 ≥10% class). These 3 patients presented a silent corticotroph tumor, persistent after surgery with slow growth, treated by radiotherapy. For 2 patients, tumor growth was controlled, and 1 patient was subsequently treated with temozolomide and a second radiotherapy (follow-up: 25, 30, and 67 months). These 3 patients presented a tumor with a low methylation score on the second PCA component (panels D and E), discriminating them from PitNETs accumulating features of aggressiveness (see also Figure S3K). Accumulation of clinicopathological features of aggressiveness was coded as defined in Figure 1.

Methylomes of 95/206 patients were analyzed using 2 approaches: unsupervised clustering to explore PitNETs molecular classification, and supervised comparisons to identify molecular signatures associated with each of the 9 clinicopathological features of aggressiveness (Figure 2A).

#### Unsupervised Methylome Classification (All Lineages)

Unsupervised clustering of all PitNETs reflected mainly lineages, with a separation between corticotroph, mammo-somato-thyrotroph, and gonadotroph PitNETs (Figure S3A). Clinicopathological features of aggressiveness, including tumor maximal diameter, Ki67 ≥ 10%, progression after radiotherapy, rapid progression, and specific death were found in specific subsets of corticotroph and mammo-somato-thyrotroph PitNETs, and absent in gonadotroph PitNETs (Figure S3A). Of note, tumor maximal diameter ≥40 mm, sphenoid invasion, cavernous invasion and metastases were not associated with any specific methylome cluster (Figure S3A).

#### Unsupervised Methylome Classification in the Corticotroph Lineage

Corticotroph PitNETs clustered in 3 groups, referred to as TPIT_m1 to TPIT_m3 (Figure 2B). Tumors with ≥40 mm maximal diameter, Ki67 ≥ 10%, progression after radiotherapy, rapid progression, temozolomide treatment, metastases, and specific death accumulated in TPIT_m1 (Figure 2B). TPIT_m2 and TPIT_m3 methylome groups included tumors with fewer clinicopathological features of aggressiveness (Figure 2B). Differentially methylated CpGs were mainly hypermethylated in TPIT_m1, and hypomethylated in TPIT_m3, irrespective of the CpG or genomic context (Figure S3B). Differentially methylated regions are reported in Table S16.

#### Clinicopathological Features of Aggressiveness with Significant Methylome Signatures (Corticotroph Lineage)

We then assessed the differential CpG methylation associated with each clinicopathological feature of aggressiveness by comparing PitNETs exhibiting the feature to those that did not. Significant methylome signatures were found for 5 clinicopathological features of aggressiveness, including Ki67 ≥ 10%, progression after radiotherapy, rapid progression, temozolomide treatment, and specific death. For tumor maximal diameter ≥40 mm, cavernous invasion, sphenoid invasion, and metastases, no specific signature was found. A merged methylome signature of aggressiveness in corticotroph PitNETs was created by merging the methylome signatures associated with the 5 clinicopathological features of aggressiveness. This signature separated a subset of tumors accumulating the significant features of aggressiveness (Figure S3C and Table S17). In this subset of PitNETs, differentially methylated CpGs showed hypermethylation, irrespective of the CpG or genomic context (Figure S3D, Table S17).

#### Unsupervised Methylome Classification in the Mammo-Somato-Thyrotroph Lineage

Mammo-somato-thyrotroph PitNETs clustered in 3 groups, referred to as PIT1_m1 to PIT1_m3 (Figure 2C). Clinicopathological features of aggressiveness were not associated with any methylome group (Figure 2C). Of note, PIT1_m3 methylome group included tumors cumulating less clinicopathological features of aggressiveness (Figure 2C). Differentially methylated CpGs were mainly hypermethylated in PIT1_m1, PIT1_m2, and hypomethylated in PIT1_m3, irrespective of the CpG or genomic context (Figure S3E). Differentially methylated regions are reported in Table S18.

#### Clinicopathological Features of Aggressiveness with Significant Methylome Signatures (Mammo-Somato-Thyrotroph Lineage)

We then assessed the differential CpG methylation associated with each clinicopathological feature of aggressiveness by comparing PitNETs exhibiting the feature to those that did not. Significant methylome signatures were found for 6 clinicopathological features of aggressiveness, including tumor maximal diameter ≥40 mm, Ki67 ≥ 10%, progression after radiotherapy, rapid progression, temozolomide treatment, and specific death. For cavernous invasion, sphenoid invasion, and metastases, no specific signature was found.

A merged methylome signature of aggressiveness in mammo-somato-thyrotroph PitNETs was created by merging methylome signatures associated with the 6 clinicopathological features of aggressiveness. This signature separated a subset of tumors accumulating the significant features of aggressiveness (Figure S3F and Table S19). In this subset of PitNETs, CpGs differentially methylated showed hypermethylation, irrespective of the CpG or genomic context (Figure S3G, Table S19).

#### Towards a Common Methylome Signature of Aggressiveness in PitNETs

Since hypermethylation was associated with aggressiveness in both corticotroph and mammo-somato-thyrotroph PitNETs, we next explored the overlap between these 2 signatures. PCA projections based on both signatures properly discriminated PitNETs accumulating features of aggressiveness, either in corticotroph or in mammo-somato-thyrotroph lineages (Figure S3H and I). Therefore, we created a common methylome signature of aggressiveness by merging these 2 signatures.

With this common signature, PitNETs accumulating clinicopathological features of aggressiveness appeared as a separate group, independently from their corticotroph and mammo-somato-thyrotroph origin (Figure S3J). Using the differentially methylated CpGs between PitNETs accumulating clinicopathological features of aggressiveness and other PitNETs, a methylome score of aggressiveness was built, corresponding to the PC1 coordinate of each tumor on the PCA projection (Figure 2D, Table S20).

#### Methylome Signatures and Proliferation in PitNETs

We next explored the relation between methylome and pathology. PitNETs with an elevated methylome score of aggressiveness were mainly associated with Ki67 ≥ 10% (Figure 2E). Discrepancies occurred in 4 patients with PitNETs showing Ki67 ≥ 10% and low methylome score of aggressiveness (Figure 2D and E).

### Gene Alteration Landscape of Aggressiveness

Gene alterations were analyzed in 146 PitNETs. Recurrent somatic gene alterations were identified in 17 genes, including *USP8*, *GNAS*, *TP53*, *LRP1B*, *CDKN2A/B*, *ATRX*, *PTEN*, *RB1*, *NOTCH2*, *KMT2C*, *ARHGAP17*, *BAG1*, *CTNNA3*, *PTPRD,* and *MEN1*. Additionally, 7 non-recurrent alterations were identified in genes previously reported in cancer or in PitNETs (Figure S4).

**Figure 3.**
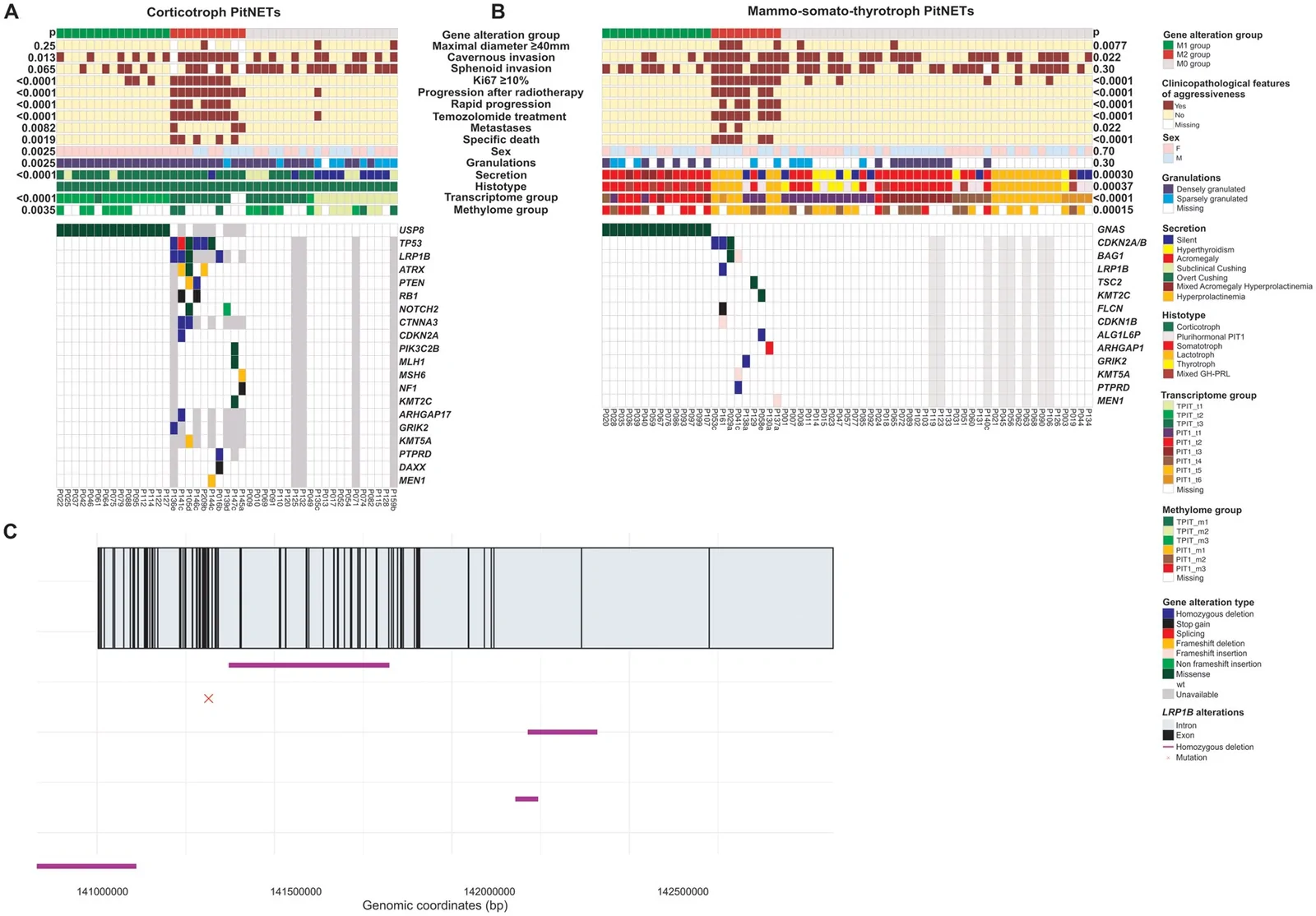
Mutational signatures of aggressiveness. (A) Classification of corticotroph tumors in 3 groups: *USP8*-mutated, cancer genes-mutated, and not mutated. (B) Classification of mammo-somato-thyrotroph PitNETs in 3 groups: *GNAS*-mutated, cancer genes-mutated, not mutated. (C) *LRP1B* gene structure. The lower part represents the observed alterations.

*LRP1B*, altered in 5 PitNETs, was the fourth most frequent alteration (Figure 3A-C). *LRP1B* is a member of the LDL receptor family, spanning over 1.9 Mb and 91 exons. All *LRP1B*-altered PitNETs displayed clinicopathological features of aggressiveness. All alterations were homozygous deletions (4/5, Figure 3C), except one likely pathogenic missense variant. Recurrent *LRP1B* alterations have been reported in several cancers, including lung cancer.^29^

Cell cycle-related genes *TP53*, *CDKN2A*, *PTEN*, and *RB1* were altered in 6, 4, 2, and 2 PitNETs, respectively, all associated with clinicopathological features of aggressiveness (Figure 3A and B). *TP53* alterations occurred only in corticotroph PitNETs, with 5 of 6 cases lacking p53 nuclear staining (data not shown). Alterations in neuroendocrine tumor genes *ATRX*, *MEN1*, and *DAXX* were also associated with clinicopathological features of aggressiveness.

In corticotroph PitNETs, *USP8* alterations were the most frequent, and associated with few clinicopathological features of aggressiveness. Other recurrent alterations, including *TP53* (*N*  =  4) and *LRP1B* (*N*  =  4), occurred in tumors enriched with clinicopathological features of aggressiveness (M2 alteration group, TPIT_t3 transcriptome group). Tumors in TPIT_t3 without these alterations did not accumulate clinicopathological features of aggressiveness (Figure 3A).

In mammo-somato-thyrotroph PitNETs, *GNAS* alterations were the most frequent and associated with a limited number of clinicopathological features of aggressiveness. Other recurrent alterations included *CDKN2A/B* (*N*  =  3) and *BAG1* (*N*  =  2). In all the tumors with clinicopathological features of aggressiveness, one of the cancer genes reported here was altered (Figure 3B).

### Longitudinal Evolution of Molecular Signatures of Aggressiveness

For 14 patients with clinicopathological features of aggressiveness, multiple samples from different surgeries were explored to evaluate the variability of molecular signatures through the course of the disease (median follow-up: 8 years, range 7 to 19 years).

#### Clonal Evolution of Aggressive PitNETs

**Figure 4.**
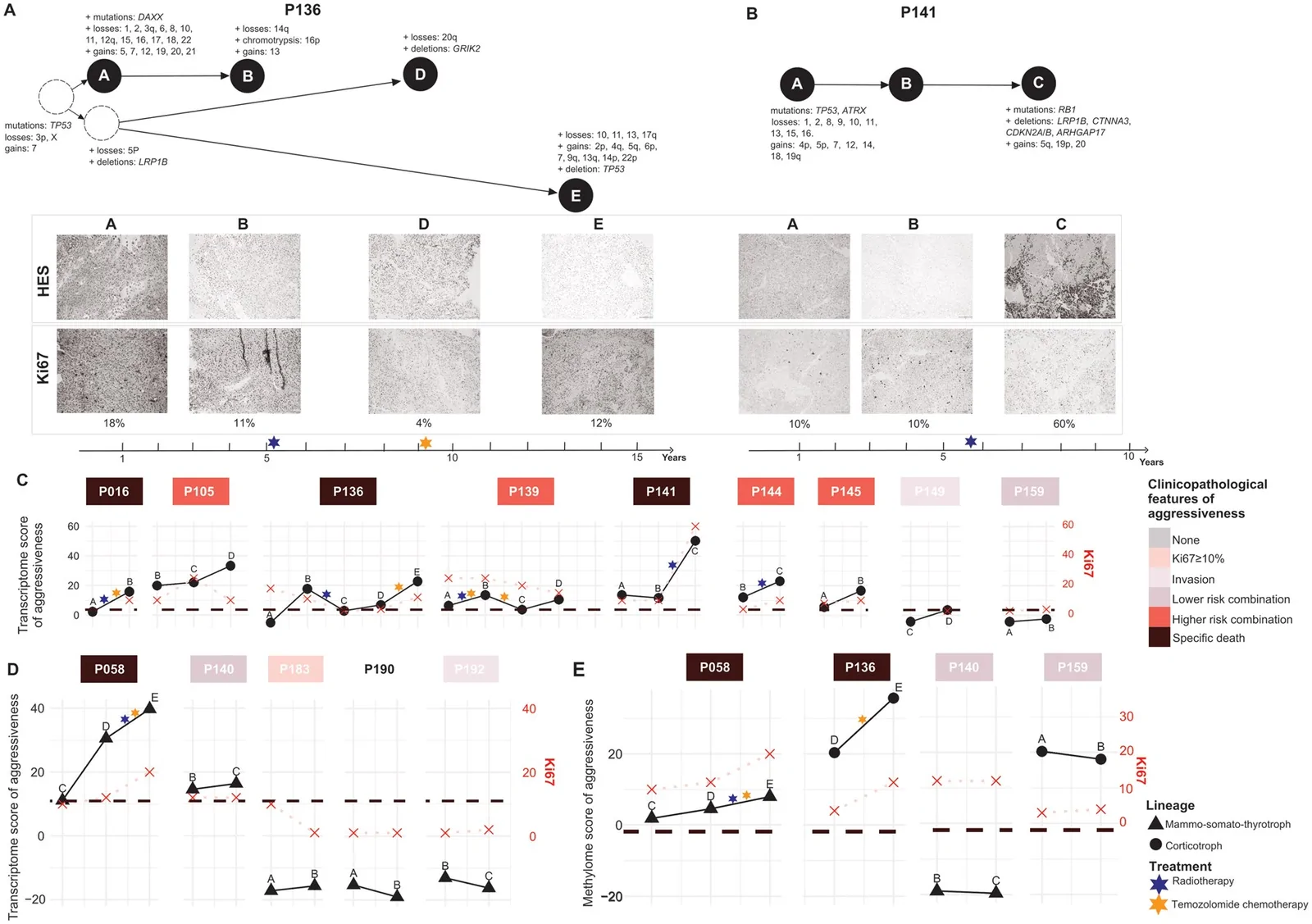
Subclonal evolution of PitNETs and its impact on transcriptome and methylome scores of aggressiveness. (A) Example of a divergent subclonal evolution. HES and Ki67 immunochemistry are represented for each sample (×200). (B) Example of a linear subclonal evolution. HES and Ki67 immunochemistry are represented for each sample (×200). (C) Transcriptome scores of aggressiveness (first Y-axis) and Ki67 (second Y-axis) in corticotroph tumors through the course of the disease. Sampling was repeated during the course of the disease in 9 patients. The brown horizontal dashed line is the transcriptome score discriminating corticotroph PitNETs accumulating significant features of aggressiveness from other corticotroph PitNETs. (D) Transcriptome scores of aggressiveness (first Y-axis) and Ki67 (second Y-axis) in mammo-somato-thyrotroph tumors through the course of the disease. Sampling was repeated during the course of the disease in 5 patients. The brown horizontal dashed line is the transcriptome score discriminating mammo-somato-thyrotroph PitNETs accumulating significant features of aggressiveness from other mammo-somato-thyrotroph PitNETs. (E) Methylome scores of aggressiveness (first Y-axis) and Ki67 (second Y-axis) in corticotroph and mammo-somato-thyrotroph tumors through the course of the disease. Sampling was repeated during the course of the disease in 4 patients. The brown horizontal dashed line is the methyolome score discriminating PitNETs accumulating significant features of aggressiveness from other PitNETs. Accumulation of clinicopathological features of aggressiveness was coded in 6 categories as detailed in Figure 1.

Somatic genetic alterations including mutations and chromosomal gains or losses were sequentially characterized through the course of the disease. A total of 17 PitNETs in 7 patients were genotyped, including 16 PitNETs with clinicopathological features of aggressiveness and one without. Clonal divergence—characterized by alterations in a subclone not found in subsequent clones—was observed in 4 patients (Figure 4A, Figure S5A-C), while no clonal divergence was observed in the 3 remaining patients (Figure 4B, Figure S5D and E).

#### Longitudinal Evolution of Transcriptome Scores of Aggressiveness

For 9 patients with corticotroph tumors, multiple samples were available. For these patients, the trajectory of transcriptome scores of aggressiveness was evaluated through the course of the disease (Figure 4C). These transcriptome trajectories shifted towards higher scores of aggressiveness. In 2 patients (P136, P139), the transcriptome trajectories were nonlinear (Figure 4C), reflecting clonal divergence in the corresponding samples (Figure 4A and Figure S5B).

In 5 patients with mammo-somato-thyrotroph tumors, transcriptome trajectories shifted towards aggressiveness only in patient P058 (Figure 4D). In this patient, the samples presented clonal divergence (Figure S5C). In the 4 other patients, transcriptome trajectories did not shift (Figure 4D).

#### Longitudinal Evolution of Methylome Scores of Aggressiveness

For 4 patients, multiple samples were available. Methylome trajectories shifted towards aggressiveness in 2 patients (P058 and P136, Figure 4E) and did not shift in 2 patients (P159 and P140, Figure 4E).

### Integrated Signatures of Aggressiveness

**Figure 5.**
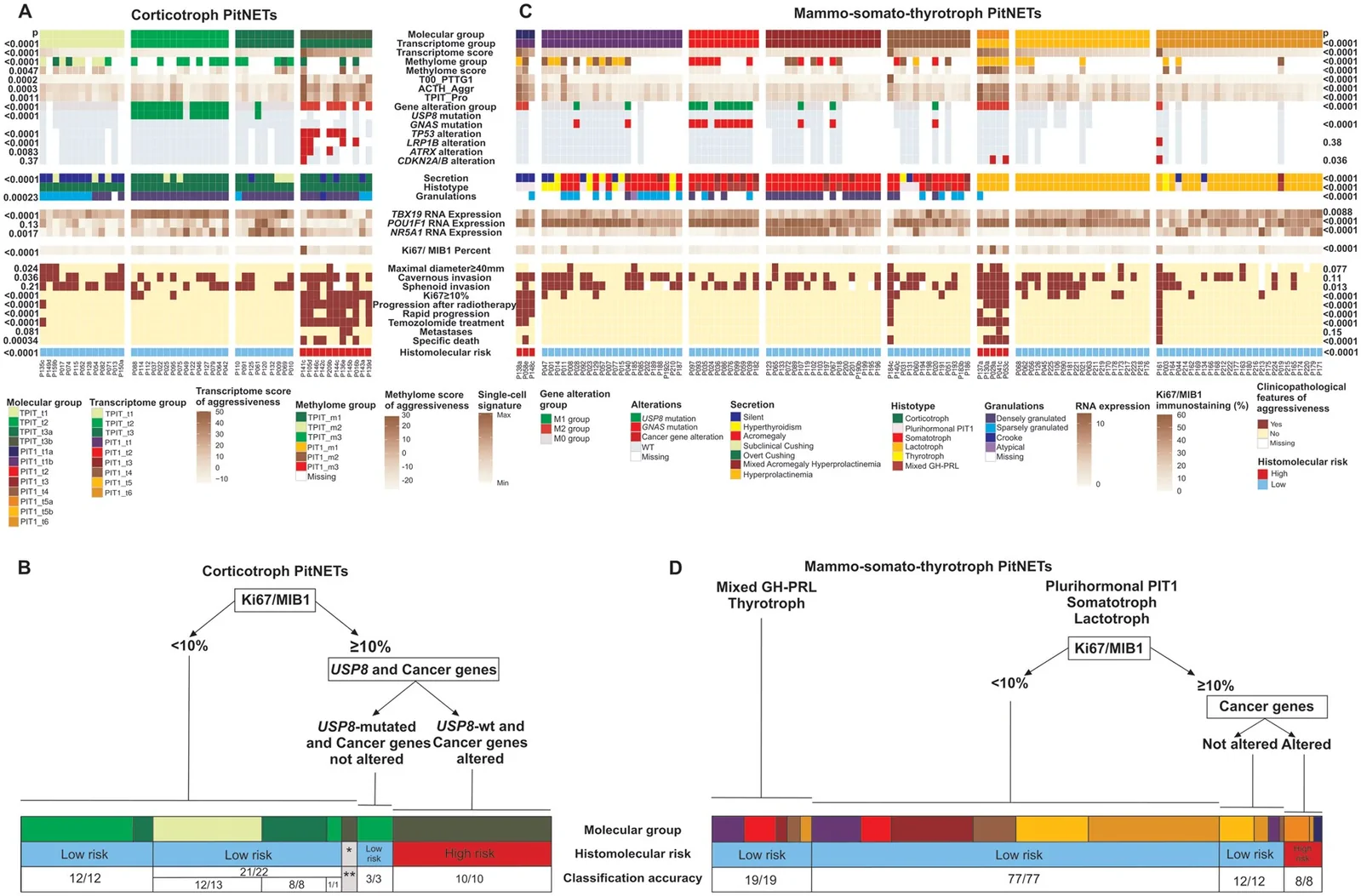
Integrated classification of PitNETs. (A) Integration of molecular and clinicopathological features of aggressiveness in corticotroph tumors. A 4-group partition is proposed to optimally classify the PitNETs, accumulating significant features of aggressiveness. (B) Proposed histomolecular workflow leading to the identification of high-risk corticotroph PitNETs. Two patients with a molecular signature of aggressiveness were not properly classified by this workflow: in a first patient (P142c), the PitNET showed a Ki67/MIB1 proliferation index <10% (*). This low proliferation index may be related to the extended necrosis in the tumor (data not shown). Due to this discrepancy, no risk of aggressiveness was inferred (**); in a second patient (P143a), no classification was proposed due to unavailable tumor DNA. (C) Integration of molecular and clinicopathological features of aggressiveness in mammo-somato-thyrotroph tumors. An 8-group partition is proposed to optimally classify the PitNETs accumulating significant features of aggressiveness. (D) Proposed histomolecular workflow leading to the identification of high-risk mammo-somato-thyrotroph PitNETs. Classification accuracy is evaluated by determining whether clinicopathological features of aggressiveness accumulate or not in the histomolecular categories.

We next gathered molecular, pathological, and clinicopathological features of corticotroph and mammo-somato-thyrotroph PitNETs. Tumors were sorted by their transcriptome group, then by their transcriptome scores of aggressiveness. In the corticotroph lineage, the TPIT_t3 group was split into 2, gathering tumors with the highest scores in TPIT_t3b, and those with the lowest scores in TPIT_t3a. This classification led to 4 molecular groups, TPIT_t1 (with gonadotroph signature), TPIT_t2 (*USP8*-mutated), TPIT_t3a (TPIT_t3 without cancer genes alterations and with low methylome scores of aggressiveness), and TPIT_t3b (TPIT_t3 with cancer genes alterations and high methylome score of aggressiveness). This classification led to high association with other molecular, pathological, and clinical features of aggressiveness (Figure 5A). Remarkably, TPIT_t3b tumors were proliferative while TPIT_t3a tumors were not (Ki67/MIB1 proliferation index, Wilcoxon *P* < .001). Overall, this classification isolated a subgroup of PitNETs accumulating both clinicopathological and molecular features of aggressiveness, referred to as high-risk corticotroph PitNETs (Figure 5A). Based on these associations, a pragmatic histomolecular workflow was proposed, combining Ki67/MIB1 proliferation index and tumor DNA sequencing, to prioritize the most accessible modalities in routine practice. This workflow led to a histomolecular risk assessment of PitNETs, optimally individualizing the subset of PiTNETs accumulating clinicopathological features of aggressiveness (Figure 5B and Figure S6A and Table S21).

Similarly, in the mammo-somato-thyrotroph lineage, the PIT1_t1 and PIT1_t5 transcriptome groups were both split into 2 subgroups, based on their transcriptome scores of aggressiveness (high scores in PIT1_t1a and PIT_t5a, and low scores in PIT1_t1b and PIT1_t5b). This classification also led to high association with other molecular, pathological, and clinical features of aggressiveness (Figure 5C). Of note, mammo-somato-thyrotroph tumors cumulating clinicopathological features of aggressiveness accumulated in the plurihormonal-PIT1 PIT1_t1a and lactotroph PIT1_t5a molecular groups. Two additional PitNETs accumulating clinicopathological features of aggressiveness were found in PIT1_t4 and PIT1_t6. All PitNETs accumulating clinicopathological features of aggressiveness were characterized by elevated proliferation (Ki67/MIB1 proliferation index, Wilcoxon *P* < .001). Overall, this classification isolated a subgroup of PitNETs accumulating both clinicopathological and molecular features of aggressiveness, referred to as high-risk mammo-somato-thyrotroph PitNETs (Figure 5C). Based on these associations, a pragmatic histomolecular workflow was also proposed. This workflow also led to a histomolecular risk assessment of PitNETs, optimally individualizing the subset of PiTNETs accumulating clinicopathological features of aggressiveness (Figure 5D and Figure S6B and Table S22).

## Discussion

Defining PitNETs’ aggressiveness remains challenging. A recent consensus statement proposed to call “aggressive” any invasive tumor with unusually rapid progression or clinically relevant growth despite optimal therapy.^21^ However, standardization of decision criteria, of treatments’ sequence, and quality is limited. Especially, the surgeon’s experience and the type of radiotherapy may induce variability. In our series, all patients were operated on by the same experienced surgical team. Regarding radiotherapy, among the 17 patients with progression after radiotherapy, 3, 4, and 10 were treated with radiosurgery, protontherapy, and fractioned radiotherapy, respectively, raising the potentially confounding impact of treatment modality. In addition, since the treatments may also impact the outcome, a poor outcome may not solely reflect the intrinsic aggressiveness. Here, we explored whether we could mitigate this limitation using unsupervised genomic classifications to better reflect the biology of PitNETs. Genomic approaches were also used to select, among all possible clinicopathological features of aggressiveness, 5 features translatable into molecular signatures. This led to a merged molecular signature of aggressiveness that defined a subgroup of PitNETs with high histomolecular risk. These tumors were invasive, multioperated, treated by radiotherapy and temozolomide, with some metastatic or lethal evolution.

In this study, no specific signature was associated with tumor invasion (cavernous or sphenoid). Accordingly, invasion has been associated with epithelial to mesenchymal transition (EMT) and extracellular matrix degradation,^30^ a feature present in a subset of cells in almost all PitNETs.^20^ Following this hypothesis, the biological feature of invasiveness may be present in all PitNETs. However, the biology of invasiveness may be more complex, potentially implicating specific spatial distribution of invasive cells, and specific tumor microenvironment patterns. Our bulk molecular analysis cannot properly catch such information, and future studies using morphological and spatial approaches should address these questions.

High proliferation is a hallmark of aggressiveness.^21^^,^^31^ Our transcriptome signatures of aggressiveness were enriched in proliferation genes. Recent single-cell studies reported proliferative cell populations, correlated with clinical aggressiveness.^19^^,^^20^ Our transcriptome scores of aggressiveness correlated with these single-cell signatures. Here, proliferation signatures differed by lineage: G1/S/M-phase in corticotroph tumors, restricted to M-phase in mammo-somato-thyrotroph. The biological meaning of these discrepant proliferation signatures remains to be determined. Finally, we observed cases with discrepant transcriptome scores of aggressiveness and Ki67/MIB1 proliferation index. These cases suggest a relevant predictive value of transcriptome scores. Beyond proliferation, other pathological features have been explored for their association with aggressiveness. Granulation patterns have been suggested as a potential marker of aggressiveness.^6^ In our cohort, specific granulation patterns were associated with specific molecular groups, but not with clinicopathological features of aggressiveness within these groups (Figure 1B and D).

*LRP1B* alterations were identified in 5 aggressive PitNETs. *LRP1B* emerges as a novel gene within PitNETs’ genomic landscape and has not been previously reported. In lung cancer, *LRP1B*-altered tumors showed elevated PD-L1 expression, immune infiltration, and better progression-free survival under immune checkpoint inhibitors.^29^ Whether *LRP1B* alterations also improve sensitivity to immune checkpoint inhibitors remains to be established in PitNETs, as well as their functional impact, especially in the context of its large spanning (91 exons over 1.9 Mb).

Beyond *LRP1B*, this study included analyses of genes known for their association with aggressiveness. We could confirm the implication of *TP53* and *ATRX* in corticotroph PitNETs, and *CDKN2A* in mammo-somato-thyrotroph PitNETs. However, gene alterations were mainly studied using targeted oncogenetic panels, limiting the ability to discover new relevant genes. Therefore, further exome (and whole genome) sequencing studies are needed, focusing on the molecular groups accumulating clinicopathological features of aggressiveness.

In this study, molecular signatures of aggressiveness were established using the latest tumor available for each patient. This choice was made *a priori*, before knowing whether a signature of aggressiveness would exist or not, to maximize the likelihood of identifying it (if this signature was restricted to late stages). However, choosing the latest sample came with 2 potential drawbacks. Firstly, the early predictive value of late signatures is not warranted. Secondly, the signatures may have been altered by “passenger” alterations accumulated during tumor evolution, including radiotherapy- or temozolomide-induced alterations.^32^^,^^33^ These potential drawbacks are mitigated by the following evidence. Firstly, in the longitudinal analyses, while repeated samplings showed a few alterations appearing as late somatic events—including *DAXX*, *ATRX*, *MEN1*, *PTEN*, *CDKN2A/B*, *RB1*, *LRP1B* alterations, a majority of somatic alterations appeared as early events and remained stable—including *TP53*, transcriptome, and methylome signatures of aggressiveness. Secondly, among the 22 patients with tumors accumulating clinicopathological features of aggressiveness, the “latest sample” corresponded to a first surgery in 5 patients. These 5 patients add to the 8 patients with longitudinal sampling showing stability of these signatures through the course of the disease. For the remaining 9 patients, the “latest sample” corresponded to late surgery samples. The latter did not cluster separately in molecular analyses. Thirdly, samples collected after radiotherapy and temozolomide treatments did not differ in terms of transcriptome, methylome, and also in terms of the number of chromosomes and gene alterations (Figure S5C). Moreover, removing these 2 features had no impact on mammo-somato-thyrotroph PitNETs classifications and molecular signatures, and a minor impact on corticotroph PitNETs transcriptome and methylome classifications and molecular signatures (Figure S7A and B). This implies that treatment-related signatures do not overcome the signatures reflecting lineage and aggressiveness. This evidence suggests that molecular characterization may provide important prognostic information irrespective of the timing of surgery. More specifically, first surgery samples may already contain the molecular prognostic information. During disease progression, re-sampling for molecular testing could be relevant to further confirm that molecular profiles do not change in their natural history or in response to treatments, particularly in tumors with changing behavior.

In our cohort, clinicopathological features of aggressiveness accumulated in subsets of corticotroph and mammo-somato-thyrotroph PitNETs, contrasting with their absence in gonadotroph (and null-cell) PitNETs. Corticotroph tumors with high proliferation clustered in TPIT_t3. In our cohort, *USP8*-mutated tumors were not aggressive. This may appear in contradiction with the reported higher rate of recurrence of Cushing’s syndrome in *USP8*-mutated PitNETs^13^ or suggest that recurrence does not always lead to aggressiveness. These findings led to a histomolecular assessment based on Ki67 proliferation index, firstly, then on *USP8* and cancer genes alteration status (*TP53*, *LRP1B*, *ATRX*), properly reflecting the outcome. Of note, in the TPIT_t3b molecular group, a subset of tumors was non-functioning. These non-functioning corticotroph tumors accumulated clinicopathological features of aggressiveness, high proliferation, and high transcriptome and methylome scores of aggressiveness. These tumors differed from the TPIT_t1 non-functioning tumors, the latter accumulating fewer clinicopathological features of aggressiveness, low proliferation, and a gonadotroph signature combined with the corticotroph lineage signature. Of note, rare instances of corticotroph PitNETs showed unfavorable evolution despite low proliferation, as previously reported.^3^

Mammo-somato-thyrotroph tumors accumulating clinicopathological features of aggressiveness were mainly lactotroph and plurihormonal PIT1, with a high proliferation index. This underlines the importance of expanding immunochemistry to the final hormones (GH, PRL, β-TSH, β-FSH, β-LH), beyond the lineage markers PIT1 and GATA3.^7^ Non-aggressive somatotroph PitNETs were associated with *GNAS* mutations or *NR5A1*/SF1 co-expression, and had low Ki67, consistent with previous studies.^14^^,^^34^ These findings led to a histomolecular risk assessment based on histological subtype, followed by Ki67, and then cancer gene mutations. Of note, *SF3B1* alterations, reported in a subset of aggressive lactotroph PitNETs,^35^ were absent in our cohort.

The new histomolecular risk assessment proposed here could have important clinical implications. Significant molecular signatures were identified for the following clinicopathological features of aggressiveness: histotype, progression after radiotherapy, rapid progression, Ki67 ≥ 10%, metastases, and specific death. External validation could help to better specify the relevance of histomolecular risk assessment to predict individual risk and whether it provides additional benefit beyond existing clinicopathological classifications. The optimal modality for molecular screening also remains to be specified. In the meantime, it could be relevant to perform a multimodal approach in these rare tumors, covering transcriptome, methylome, and targeted tumor DNA sequencing. The latter could be derived from targeted oncopanels (commonly including *TP53*, *ATRX*, *CDKN2A*, *MEN1*, *DAXX*), enriched with PitNETs’ specific genes (*USP8*, *GNAS*, *SF3B1*, and *LRP1B*). Following our proposed histomolecular workflow, corticotroph and mammo-somato-thyrotroph tumors with Ki67 ≥ 10% could be proposed for a molecular evaluation, after a first surgery and following ones. Low-risk tumors may require lighter surveillance, high-risk tumors closer monitoring, and earlier aggressive treatments. Such a proactive approach was recently proposed in a recent recommendation.^21^ Validating this histomolecular workflow could lead to the subsequent question of additional treatments immediately after surgery for high-risk patients (potentially radiotherapy, either alone or combined with temozolomide) without waiting for progression.

In conclusion, this study refines the concept of aggressiveness in PitNETs with a molecular definition and identifies recurrent alterations in *LRP1B—*a novel targetable gene in PitNETs genomic landscape. The impact of subclonal evolution on the histomolecular risk seems limited but requires external validation. High-risk PitNETs with unfavorable outcome can be individualized according to our histomolecular risk assessment workflow.

## Supplementary Material

noag074_Supplementary_Data

## Data Availability

Anonymized mRNA sequencing data have been deposited at the European Genome-phenome Archive (EGA) under accession number EGAC00001001196. Methylation values table is available on Zenodo (10.5281/zenodo.17407564).
