# Supplementary material for: Genomic characterization of aggressiveness in pituitary neuroendocrine tumors
Source: Neuro Oncol. 2026 Aug 6;28(9):2166–79. doi: 10.1093/neuonc/noag074 (PMC13550688; doi:10.1093/neuonc/noag074)
Supplement: Supplementary file 1 [file noag074_supplementary_data.zip › Supplementary_Material_Corrected_Clean_260312.docx]

Supplementary Materials for:

Genomic characterization of aggressiveness in pituitary neuroendocrine tumors (PitNETs)

**Authors:** Nesrine Benanteur*^1^, Chiara Villa^1,2^ , Fabio Bioletto^1,3^, Maria Francesca Birtolo^1,4^, Daniel De Murat^1^, Julien Masliah-Planchon^5^, Victor Gravrand^1^, Inès Lebib^1^, Mounia Benkhellat^1^, Robin Boezennec^1^, Stéphanie Allassonnière^6^, Franck Letourneur^7^, Yoann Martin^1,7^, Fidéline Bonnet Serrano^1,8^, Julian Jacob^9^, Valentin Calugaru^10^, Maxime Barat^1,11^, Anthony Dohan^1,11^, Jennifer Arrondeau^12^, Olivier Huillard^13^, Marie-Laure Raffin-Sanson^14,15^, Jean François Emile^16^, Gérald Raverot^17,18^, Rossella Libé^1,19^, Laurence Guignat^19^, Cyril Garcia^20^, Lionel Groussin^1,19^, Xavier Bertagna^1,19^, Stephan Gaillard^1^, Jérôme Bertherat^1,19^, Anne Jouinot^1^, Bertrand Baussart^1,21^, Guillaume Assié*^1,19^

**Affiliations:**

1. CNRS UMR8104, INSERM U1016, Institut Cochin, Université Paris Cité; Paris 75014, France (NB, CV, FB, MFB, DdM, VG, IL, MoB, RB, YM, FBS, MaB, RL, LiG, XB, SG, JB, AJ, BB, GA).

2. Department of Pathology, Hôpital Lariboisière, Assistance Publique-Hôpitaux de Paris; Paris 75010, France. (CV)

3. Division of Endocrinology, Diabetes and Metabolism, Department of Medical Sciences, University of Turin;10126 Turin, Italy.(FB)

4. Department of Biomedical Sciences, Humanitas University; Pieve Emanuele Milan 20090, Italy.(MFB)

5. Department of Diagnostic and Theranostic Medicine, Somatic Genetics Unit, Institut Curie, Paris-Science Lettres University; Paris, France.(JMP)

6. Université Paris Cité, Unité Mixte de Recherche S1138, Institut national de recherche en sciences et technologies du numérique, Sorbonne University; Paris, France.(SA)

7. INSERM U1016, Institut Cochin, Plate-Forme Séquençage et Génomique (Genom'IC); Paris 75014, France.(FL, YM)

8. Hormonology Department, Cochin Hospital; Paris F-75014, France. (FBS)

9. Department of Radiation Oncology, Hôpital La Pitié-Salpêtrière, Assistance Publique-Hôpitaux de Paris; 75013 Paris, France.(JJ)

10. Department of Radiation Oncology, Paris/Saint-Cloud/Orsay, Institut Curie, PSL Research University; 75005 Paris, France.(VC)

11. Department of Radiology, Hôpital Cochin, Assistance Publique-Hôpitaux de Paris; 75014 Paris, France.(MB, AD)

12. Department of Clinical Oncology, Hôpital Cochin, Assistance Publique-Hôpitaux de Paris; 75014 Paris, France.(JA)

13. Medical Oncology Department, Université Paris Cité, Institut du Cancer Paris CARPEM, AP-HP, Hôpital Cochin-Port Royal; 75014, Paris, France.(OH)

14. Department of Endocrinology, Diabetology, and Nutrition, Ambroise Paré University Hospital, Assistance Publique-Hôpitaux de Paris; F-92100 Boulogne Billancourt, France. (MLRS)

15. EA4340, University of Versailles Saint-Quentin-en-Yvelines, UFR of Health Sciences Simone Veil; F-78423 Montigny-le-Bretonneux, France. (MLRS)

16. Department of Pathology, Hôpital Ambroise Paré, Assistance Publique-Hôpitaux de Paris; Boulogne-Billancourt 92100, France. (JFE)

17. Cancer Research Center of Lyon, Inserm U1052, CNRS UMR5286, Lyon 1 University; Lyon, France.(GR)

18. Endocrinology Department, Reference Center for Rare Pituitary Diseases HYPO, "Groupement Hospitalier Est" Hospices Civils de Lyon; Bron, France. (GR)

19. Department of Endocrinology, Hôpital Cochin, Reference and Competence Center for Rare Adrenal Diseases and Rare Pituitary Diseases, Assistance Publique-Hôpitaux de Paris; 75014 Paris, France. (RL, LiG, LaG, XB, JB, GA)

20. Service D'Endocrinologie, Hôpital National D'Instruction des Armées Bégin; 94160 Saint-Mandé, France. (CG)

21. Department of Neurosurgery, Hôpital Lariboisière, Assistance Publique-Hôpitaux de Paris; Paris 75010, France. (BB)

***Corresponding authors: Guillaume Assié,** [**guillaume.assie@aphp.fr**](mailto:guillaume.assie@aphp.fr)**; Nesrine Benanteur, nesrine.benanteur@gmail.com**

**Journal: *Neuro-Oncology***

**Date: 11th March 2026**

Supplementary methods

**Transcriptome analyses**

Frozen samples were selected directly by the surgeons during intervention, targeting the most representative fragments with minimal contamination and necrosis. These samples were directly snap-frozen in liquid nitrogen and stored at -80°C. Total RNA was then extracted using RNAble (Eurobio) and purified with RNeasy columns (Qiagen). FFPE samples were selected by an expert neuropathologist. For each sample, 5 to 10 tissue sections (10-15 µm thick) were cut from FFPE blocks, depending on tumoral tissue availability. Total RNA was then extracted using the RNeasy DSP FFPE Kit (Qiagen).

RNA was quantified using Qubit fluorometer (Thermo Fisher Scientific) and RNA quality assessed by electrophoresis on a Bioanalyser 2100 (Agilent Technologies).

Library preparation was performed at the Genomics Platform of the Institut Cochin. For frozen samples, library preparation was performed following the TruSeq Stranded mRNA protocol (Illumina), starting from 1 μg of high-quality total RNA. Paired end (2 × 75 bp) sequencing was carried out on a Nextseq 500 platform (Illumina). For FFPE samples, library preparation was performed following the QuantSeq 3’ mRNA-Seq protocol (Lexogen), starting from 1 to 500 ng RNA. Single read sequencing (1 ×75bp) was performed on a NextSeq 550 platform (Illumina).

FASTQ sequences were aligned on the GRCh37 human reference genome with STAR^1^ (v2.7.3a). Samples quality was assessed on raw read counts. Only samples with at least 30% of transcripts detected were retained for downstream analysis. Read counts were subsequently normalized using the RUVg function from RUVSeq R package^2^ (v0.99.1), with k=1, and using a list of housekeeping genes published by Eiseinberg et al.^3^. Genes were then filtered by standard deviation in order to select the top most variable for unsupervised classifications.

Transcriptome groups were defined through unsupervised non-negative matrix factorization consensus clustering with 500 iterations, implemented with the NMF R package^4^ (v0.28.0). The best classification was chosen from the optimal number of ranks (from 2 to 10) and the optimal number of genes (top 500, 1000, 1500, 2000, 2500, 3000 most variable genes), leading to the highest cophenetic coefficient and silhouette width score. Unsupervised hierarchical clustering was then performed from the NMF coefficient matrix using Euclidean distance, and plots were subsequently generated using Pheatmap R package^5^ (v1.0.12). Differentially expressed genes between transcriptomic groups defined by the NMF were identified using Limma.

Canonical transcriptomic signatures for each of the 5 pituitary endocrine cell type (corticotroph, lactotroph, thyrotroph, gonadotroph, and somatotroph) were determined by averaging the gene expression data of the available samples for each tumor type. For each tumor, the relative contribution of the 5 cell types was then estimated by linear regression analysis.

Differentially expressed genes associated with each feature of aggressiveness were determined independently for corticotroph and mammo-somato-thyrotroph PitNETs, using Limma. Gene-set enrichment analyses were carried out using clusterProfiler (v4.14.6) R package, using the gene ontology annotations (GO All)^6,7^. Volcano plots were generated using the EnhancedVolcano^8^ (v1.24.0) R package.

**Methylome analyses**

Raw data were processed with Minfi^9^ (v1.24.0). Probes were filtered using default parameters and beta-values were extracted, then normalized with the ‘‘Funnorm’’ function^10^. Adjacent CpGs were collapsed into CpG features, using the ‘‘cpgCollapse’’ function with default parameters. Beta-values were converted into M-values. The most variable CpGs were selected for subsequent analyses.

Unsupervised hierarchical clustering was used to classify PitNETs using Euclidean distance. Different partitions were tested using the “cutree” function ranging from 2 to 10 clusters, applied to both the PitNET tree and the CpG tree. Each double partition led to a heatmap of CpG blocks. The optimal partition corresponded to the most contrasted heatmap (with maximal inter-block variability and minimal intra-block variability).

Differentially methylated CpGs associated with each feature of aggressiveness were determined independently for corticotroph and mammo-somato-thyrotroph PitNETs, using Limma. Enrichment analyses were performed using the IlluminaHumanMethylationEPICanno.ilm10b4.hg19 R Package^11^ (v0.16.1).

**Statistical analyses**

Calculations were performed using R statistical software (v4.4.3). Comparisons between groups were performed with Student T, Wilcoxon Mann Whitney and Kruskal–Wallis tests for quantitative variables, and with Chi-squared or Fisher’s exact test for qualitative variables. All p values were two-sided, and the level of significance was set at p < 0.05. Correlations were computed in R using the cor() function with Pearson’s method,

Group comparison in transcriptome and methylome were performed with the Limma R package^12^ (v3.62.2), with Benjamini-Hochberg correction for multiple testing.

Principal component analyses (PCA) were carried out with stats^13^ (v3.6.2) and ggplot2^14^ (v3.5.2) R packages.

1.	Dobin A, Davis CA, Schlesinger F, et al. STAR: ultrafast universal RNA-seq aligner. *Bioinformatics*. 2013;29(1):15-21. doi:10.1093/bioinformatics/bts635

2.	Risso D, Ngai J, Speed TP, Dudoit S. Normalization of RNA-seq data using factor analysis of control genes or samples. *Nat Biotechnol*. 2014;32(9):896-902. doi:10.1038/nbt.2931

3.	Eisenberg E, Levanon EY. Human housekeeping genes, revisited. *Trends Genet*. 2013;29(10):569-574. doi:10.1016/j.tig.2013.05.010

4.	A flexible R package for nonnegative matrix factorization - PubMed. Accessed December 17, 2025. https://pubmed.ncbi.nlm.nih.gov/20598126/

5.	Kolde R. pheatmap: Pretty Heatmaps. Published online October 24, 2010:1.0.13. doi:10.32614/CRAN.package.pheatmap

6.	Wu T, Hu E, Xu S, et al. clusterProfiler 4.0: A universal enrichment tool for interpreting omics data. *Innovation (Camb)*. 2021;2(3):100141. doi:10.1016/j.xinn.2021.100141

7.	Gene Ontology Consortium. The Gene Ontology resource: enriching a GOld mine. *Nucleic Acids Res*. 2021;49(D1):D325-D334. doi:10.1093/nar/gkaa1113

8.	EnhancedVolcano. Bioconductor. Accessed December 18, 2025. http://bioconductor.org/packages/EnhancedVolcano/

9.	Aryee MJ, Jaffe AE, Corrada-Bravo H, et al. Minfi: a flexible and comprehensive Bioconductor package for the analysis of Infinium DNA methylation microarrays. *Bioinformatics*. 2014;30(10):1363-1369. doi:10.1093/bioinformatics/btu049

10.	Fortin JP, Triche TJ, Hansen KD. Preprocessing, normalization and integration of the Illumina HumanMethylationEPIC array with minfi. *Bioinformatics*. 2017;33(4):558-560. doi:10.1093/bioinformatics/btw691

11.	IlluminaHumanMethylationEPICanno.ilm10b4.hg19. Bioconductor. Accessed December 18, 2025. http://bioconductor.org/packages/IlluminaHumanMethylationEPICanno.ilm10b4.hg19/

12.	Ritchie ME, Phipson B, Wu D, et al. limma powers differential expression analyses for RNA-sequencing and microarray studies. *Nucleic Acids Res*. 2015;43(7):e47. doi:10.1093/nar/gkv007

13.	stats package - RDocumentation. Accessed December 18, 2025. https://www.rdocumentation.org/packages/stats/versions/3.6.2

14.	Create Elegant Data Visualisations Using the Grammar of Graphics. Accessed December 18, 2025. https://ggplot2.tidyverse.org/

Supplementary Figures

**Figure S1**: Processing and quality control of samples

**(A)** Interaction diagram of the samples and the omics approaches.

**(B)** Assessment of the tumor cells content by the proportion of each canonical pituitary cell type signature, using the transcriptome of 205 samples. Median tumor purity was ≥70% in all groups but t1. For t1, the method for inferring tumor cells content was impacted by the dual corticotroph/gonadotroph differentiation, increasing the gonadotroph signature in these corticotroph tumors (purity is probably ≥80% in this group).

**(C)** Assessment of the tumor cells content by the allelic ratios of chromosomal alterations, using the SNP array of 107 samples. Median purity was ≥90% in all groups, including t1 transcriptome group.

**Figure S2**: Transcriptome signatures of aggressiveness

**(A)** Unsupervised classification of all PitNETs; the most robust partition was obtained with 6 NMF ranks and the 3000 most variable genes.

**(B)** Principal component analysis projection of corticotroph PitNETs using the corticotroph merged transcriptome signature of aggressiveness.

**(C)** Differentially expressed genes between corticotroph PitNETs accumulating significant features of aggressiveness and other PitNETs; vulcano plot representation (left panel); gene set enrichment (right panel).

**(D)** Principal component analysis projection of mammo-somato-thyrotroph PitNETs using the mammo-somato-thyrotroph merged transcriptome signature of aggressiveness.

**(E)** Differentially expressed genes between mammo-somato-thyrotroph PitNETs accumulating significant features of aggressiveness and other PitNETs; vulcano plot representation (left panel); gene set enrichment (right panel).

**(F)** Principal component analysis projection of both corticotroph (circles) and mammo-somato-thyrotroph (triangles) tumors using the corticotroph merged transcriptome signature of aggressiveness.

**(G)** Principal component analysis projection of both corticotroph (circles) and mammo-somato-thyrotroph (triangles) tumors using the mammo-somato-thyrotroph merged transcriptome signature of aggressiveness.

**(H)** Correlation plots between the transcriptome score of aggressiveness of corticotroph tumors and T00_PTTG1 single cell signature from Yan et al., 2024 (19); ACTH_agg single cell signature from Su et al., 2025 (20) ; and TPIT_pro single cell signature from Su et al., 2025 (20).

**(I)** Correlation plots between the transcriptome score of aggressiveness of mammo-somato-thyrotroph tumors and T00_PTTG1 single cell signature from Yan et al., 2024 (19); ACTH_agg single cell signature from Su et al., 2025 (20); and TPIT_pro single cell signature from Su et al., 2025 (20).

Accumulation of clinicopathological features of aggressiveness was coded in 6 categories as detailed in Figure 1.

**Figure S3:** Methylome signatures of aggressiveness

**(A)** Unsupervised classification of all PitNETs; the most robust partition was obtained with the 6000 most variable CpGs.

**(B)** Distribution of differentially methylated CpGs associated with corticotroph methylome groups.

**(C)** Principal component analysis projection of corticotroph PitNETs using the corticotroph merged methylome signature of aggressiveness.

**(D)** Distribution of differentially methylated CpGs between PitNETs accumulating significant features of aggressiveness and other PitNETs in corticotroph tumors.

**(E)** Distribution of differentially methylated CpGs associated with mammo-somato-thyrotroph methylome groups.

**(F)** Principal component analysis projection of mammo-somato-thyrotroph PitNETs using the mammo-somato-thyrotroph merged methylome signature of aggressiveness.

**(G)** Distribution of differentially methylated CpGs between PitNETs accumulating significant features of aggressiveness and PitNETs and other PitNETs in mammo-somato-thyrotroph tumors.

**(H)** Principal component analysis projection of both corticotroph (circles) and mammo-somato-thyrotroph (triangles) tumors using the corticotroph merged methylome signature of aggressiveness.

**(I)** Principal component analysis projection of both corticotroph (circles) and mammo-somato-thyrotroph (triangles) tumors using the mammo-somato-thyrotroph merged transcriptome signature of aggressiveness.

**(J)** Principal component analysis projection of corticotroph (circles) and mammo-somato-thyrotroph (triangles) tumors using the common methylome signature of aggressiveness.

**(K)** Boxplot representation in corticotroph and mammo-somato-thyrotroph tumors of the PC2 methylome score depending on Ki67/MIB1 proliferaton index as defined by pathology.

Accumulation of clinicopathological features of aggressiveness was coded in 6 categories as detailed in Figure 1.

**Figure S4:** Gene alterations classification of all PitNETs.

**Figure S5:** Subclonal evolution of PitNETs

**(A)** Subclonal evolution of patient P105.

**(B)** Subclonal evolution of patient P139.

**(C)** Subclonal evolution of patient P058.

**(D)** Subclonal evolution of patient P144.

**(E)** Subclonal evolution of patient P016.

HES and Ki67 immunostaining are provided for each sample (x200, lower panels).

**Figure S6:** Association between transcriptome, methylome, gene alteration and molecular groups with clinicopathological features of aggressiveness

**(A**) Associations in corticotroph PitNETs.

**(B**) Associations in mammo-somato-thyrotroph PitNETs.

**Figure S7:** Impact of progression after radiotherapy and temozolomide treatment on classifications

**(A)** Transcriptome score of aggressiveness in corticotroph PitNETs after removing the “progression after radiotherapy” and “temozolomide treatment” features: the projection of tumors is comparable with the projection in Figure 1C (before removing these two features). The transcriptome signature after removing “progression after radiotherapy” and “temozolomide treatment” shares 592 genes among the 671 genes of the signature before removing these two features (88%).

**(B)** Methylome score of aggressiveness in corticotroph and mammo-somato-thyrotroph PitNETs after removing the “progression after radiotherapy” and “temozolomide treatment” features: the projection of tumors is comparable with the projection in Figure 2C (before removing these two features). The transcriptome signature after removing “progression after radiotherapy” and “temozolomide treatment” shares 1360 CpGs among the 1393 CpGs of the initial signature (98%).

**(C)** Genome alteration burden before and after temozolomide treatment.

Accumulation of clinicopathological features of aggressiveness was coded in 6 categories as detailed in Figure 1.

Supplementary Tables

Table S1: Antibody features and experimental conditions for immunohistochemistry analyses.

| **Antibody** | **Provider** | **Clone** | **Host species** | **Dilution** |
| --- | --- | --- | --- | --- |
| ACTH | Bio SB | BSB25 | mouse | 1/1000 |
| Beta-LH | DAKO | C93 | mouse | 1/50 |
| Beta-TSH | DAKO | 42 | mouse | 1/300 |
| Beta-FSH | Cell Marque | EP257 | rabbit | 1/200 |
| Prolactine | Roche | 5268249001 | rabbit poly | 1/300 |
| hGH | Agilent | A057001-2 | rabbit poly | 1/3000 |
| CAM 5.2 | BECTON | CAM5.2 | mouse | pre-diluted |
| PIT-1 | NOVUS BIO | POU1-F1 | rabbit | 1/500 |
| T-Pit | ATLAS AB | CL6251 | mouse | 1/200 |
| SF-1 | ABCAM | EPR19744 | rabbit | 1/500 |
| GATA3 | ROCHE | GATA3 | mouse | pre-diluted |
| Ki67 | DAKO | MIB-1 | mouse | 1/50 |
| p53 | DAKO | DO-7 | mouse | 1/50 |
| ATRX | Bio SB | BSB108 | mouse | 1/200 |

**Table S2:** Association between clinical features of aggressiveness and other features in the cohort of 206 PitNETs.

**Table S3:** Genes coefficients contributing to the 6 NMF ranks of the transcriptome classification of all PitNETs.

**Table S4:** Genes coefficients contributing to the 2 NMF ranks of the transcriptome classification of corticotroph PitNETs.

**Table S5:** Differentially expressed genes in corticotroph groups.

**Table S6:** Gene set enrichments among differentially expressed genes in corticotroph groups.

**Table S7:** Differentially expressed genes associated with each clinical feature of aggressiveness in corticotroph tumors. The “accumulated significant features” columns refer to the comparison between PitNETs accumulating the significant features of aggressiveness and other PitNETs.

**Table S8:** Gene set enrichment among differentially expressed genes between corticotroph PitNETs accumulating the significant features of aggressiveness and other PitNETs.

**Table S9:** Genes coefficients contributing to the 4 NMF ranks of the transcriptome classification of mammo-somato-thyrotroph PitNETs.

**Table S10:** Differentially expressed genes in mammo-somato-thyrotroph groups.

**Table S11:** Gene set enrichments among differentially expressed genes in mammo-somato-thyrotroph groups.

**Table S12:** Differentially expressed genes associated with each clinical feature of aggressiveness in mammo-somato-thyrotroph tumors. The “accumulated significant features” columns refer to the comparison between PitNETs accumulating the significant features of aggressiveness and other PitNETs.

**Table S13:** Gene set enrichment among differentially expressed genes between mammo-somato-thyrotroph PitNETs accumulating the significant features of aggressiveness and other PitNETs.

**Table S14:** Correlations between the transcriptome score of aggressiveness and single cell signatures in corticotroph tumors. Single cell signatures were obtained from Zhang et al., 2023 (28), Yan et al. (19), 2024 and Su et al., 2025 (20).

**Table S15:** Correlations between the transcriptome score of aggressiveness and single cell signatures in mammo-somato-thyro tumors. Single cell signatures were obtained from Zhang et al., 2023 (28), Yan et al. (19), 2024 and Su et al., 2025 (20).

**Table S16:** Differentially methylated regions between groups in corticotroph tumors.

**Table S17:** Differentially methylated CpGs associated with each clinical feature of aggressiveness in corticotroph tumors. The “accumulated significant features” columns refer to the comparison between PitNETs accumulating the significant features of aggressiveness and other PitNETs.

**Table S18:** Differentially methylated regions between groups in mammo-somato-thyrotroph tumors.

**Table S19:** Differentially methylated CpGs associated with each clinical feature of aggressiveness in mammo-somato-thyrotroph tumors. The “accumulated significant features” columns refer to the comparison between PitNETs accumulating the significant features of aggressiveness and other PitNETs.

**Table S20:** Differentially methylated CpGs between PitNETs (corticotroph and mammo-somato-thyrotroph) accumulating the significant features of aggressiveness and other PitNETs.

**Table S21**: Contingency table showing the distribution of clinicopathological features of aggressiveness across risk groups in corticotroph PitNETs

**Table S22**: Contingency table showing the distribution of clinicopathological features of aggressiveness across risk groups in mammo-somato-thyrotroph PitNETs
